# The proto-oncogene c-Src and its downstream signaling pathways are inhibited by the metastasis suppressor, NDRG1

**DOI:** 10.18632/oncotarget.3316

**Published:** 2015-04-10

**Authors:** Wensheng Liu, Fei Yue, Minhua Zheng, Angelica Merlot, Dong-Hun Bae, Michael Huang, Darius Lane, Patric Jansson, Goldie Yuan Lam Liu, Vera Richardson, Sumit Sahni, Danuta Kalinowski, Zaklina Kovacevic, Des. R. Richardson

**Affiliations:** ^1^ Department of General Surgery, Ruijin Hospital, Shanghai Jiao Tong University School of Medicine, Shanghai 200025, P.R.China; ^2^ Molecular Pharmacology and Pathology Program, Department of Pathology and Bosch Institute, University of Sydney, Sydney, New South Wales 2006, Australia

**Keywords:** NDRG1, metastasis suppressor, c-Src, cell migration

## Abstract

N-myc downstream regulated gene-1 (NDRG1) is a potent metastasis suppressor that plays a key role in regulating signaling pathways involved in mediating cancer cell invasion and migration, including those derived from prostate, colon, *etc*. However, the mechanisms and molecular targets through which NDRG1 reduces cancer cell invasion and migration, leading to inhibition of cancer metastasis, are not fully elucidated. In this investigation, using NDRG1 over-expression models in three tumor cell-types (namely, DU145, PC3MM and HT29) and also NDRG1 silencing in DU145 and HT29 cells, we reveal that NDRG1 decreases phosphorylation of a key proto-oncogene, cellular Src (c-Src), at a well-characterized activating site (Tyr416). NDRG1-mediated down-regulation of EGFR expression and activation were responsible for the decreased phosphorylation of c-Src (Tyr416). Indeed, NDRG1 prevented recruitment of c-Src to EGFR and c-Src activation. Moreover, NDRG1 suppressed Rac1 activity by modulating phosphorylation of a c-Src downstream effector, p130Cas, and its association with CrkII, which acts as a “molecular switch” to activate Rac1. NDRG1 also affected another signaling molecule involved in modulating Rac1 signaling, c-Abl, which then inhibited CrkII phosphorylation. Silencing NDRG1 increased cell migration relative to the control and inhibition of c-Src signaling using siRNA, or a pharmacological inhibitor (SU6656), prevented this increase. Hence, the role of NDRG1 in decreasing cell migration is, in part, due to its inhibition of c-Src activation. In addition, novel pharmacological agents, which induce NDRG1 expression and are currently under development as anti-metastatic agents, markedly increase NDRG1 and decrease c-Src activation. This study leads to important insights into the mechanism involved in inhibiting metastasis by NDRG1 and how to target these pathways with novel therapeutics.

## INTRODUCTION

The metastatic spread of primary cancers contributes to approximately 90% of all cancer deaths [1]. However, understanding of the mechanisms modulating local migration, invasion and the formation of metastases remains poorly characterized at the molecular level [2]. In fact, there are many proto-oncogenes that play key roles in regulating cellular signaling resulting in cancer cell migration and invasion, with cellular Src (c-Src) being vital in modulating these processes [3].

c-Src is one of the most well-characterized proto-oncogenes and non-receptor protein tyrosine kinases [4], which can be activated by key receptor tyrosine kinases (*e.g*., epidermal growth factor receptor (EGFR) and platelet-derived growth factor receptor (PDGFR)) and protein tyrosine phosphatases (*e.g*., PTP1B and PTP-PEST) [5, 6]. Once activated, c-Src can interact with various substrates and key effectors of oncogenic signaling cascades, which affects various cellular functions, such as proliferation, cell cycle, adhesion, differentiation, and migration [3, 4]. In fact, c-Src is known to be over-expressed and/or hyper-activated in a wide variety of human cancers, including colon and prostate [7, 8]. Aberrant c-Src expression and/or activity are believed to play a vital role in cell transformation, the epithelial-to-mesenchymal transition (EMT), cancer development and progression [3, 9].

A number of oncogenic signaling molecules and pathways are involved in c-Src-mediated cancer cell invasion and migration, including the chicken tumor virus No.10 (CT10) regulator of kinase (Crk)-associated substrate (p130Cas), Abelson murine leukemia viral oncogene homolog 1 (ABL1, also known as c-Abl), paxillin, focal adhesion kinase (FAK), as well as PI3K-Akt, Ras-Raf-MEK-ERK1/2-MAPK, STAT3-IL8-VEGF pathways, *etc.* [3, 10]. The c-Src-substrate interaction induces the efficient phosphorylation and activation of substrates, which in turn serves to initiate downstream signaling involving p130Cas, *etc.* [10], and regulates cytoskeleton organization, cell adhesion, cell migration and invasion.

**Figure 1:**
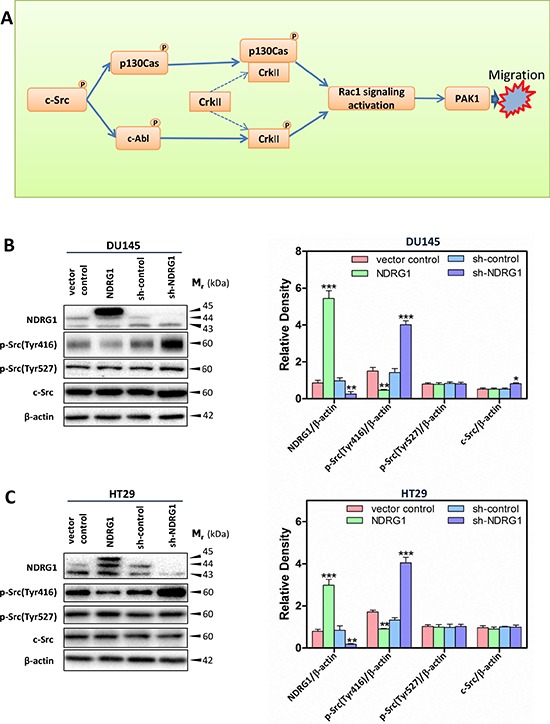
Schematic diagram illustrating the c-Src signaling pathway assessed herein (A) and immunoblots revealed that NDRG1 expression inhibited c-Src phosphorylation (Tyr416) in DU145 cells (B) and HT29 cells (C) (B, C) Whole-cell lysates were prepared, and immunoblotting was performed to determine the effect of NDRG1 expression on levels of phosphorylated (p-) c-Src (p-Src(Tyr416) and p-Src(Tyr527)) and total c-Src compared to that of the relative control cells (vector control and sh-control). Blots are representative of 3–5 experiments. Densitometric analysis is expressed relative to the β-actin loading control. Data show the mean ± S.D. (3–5 experiments); *p < 0.05; **p < 0.01; ***p < 0.001, relative to vector control or sh-control cells, as appropriate.

The phosphorylation and activation of p130Cas is one of the key initial events in downstream c-Src signaling (Figure 1A) [11]. Interestingly, phosphorylation of p130Cas promotes its binding to CrkII, which subsequently recruits DOCK180, leading to the activation of the Rho family GTPase, Ras-related C3 botulinum toxin substrate 1 (Rac1; Figure 1A) [12, 13]. Additionally, c-Src can also activate c-Abl, which plays an important role in regulating cell motility in response to PDGF [14]. In fact, c-Abl interacts with and phosphorylates CrkII at Tyr221, which is required for Rac1 signaling activation that is involved in cytoskeleton dynamics, adhesion and cell migration (Figure 1A) [15, 16]. Rac1 plays a crucial role in regulating cancer cell motility by virtue of cycling between inactive GDP-bound and active GTP-bound forms (*i.e*., GTP-Rac1) [17]. Aberrant Rac1 activation associated with c-Src activation, contributes to the development and progression of a variety of cancers, and is accompanied with poor prognosis, cancer invasion and metastasis [18].

While c-Src signaling can promote cancer metastasis, there are several proteins that can act as metastasis suppressors [19]. In fact, the expression of one of these molecules, namely N-myc downstream-regulated gene 1 (NDRG1), which is also known as Cap43, could be induced by hypoxia [20] and was negatively correlated with cancer grade and metastasis [21–24]. NDRG1 is predominantly a cytosolic, ubiquitously expressed protein [25], which has been shown to play diverse roles in cellular signaling, affecting transforming growth factor-β (TGF-β) [26], protein kinase B (AKT) [26], nuclear factor kappa-light-chain-enhancer of activated B cells (NF-κB) [27] and WNT signaling pathways [28].

Interestingly, our recent investigations have revealed that NDRG1 inhibits a crucial step in metastasis, namely the TGF-β-induced EMT, which occurs by the ability of NDGR1 to maintain E-cadherin and β-catenin at the cell membrane, leading to decreased vimentin expression and suppression of cell migration and invasion [29]. Furthermore, it has also been demonstrated that NDRG1 inhibits phosphorylation and nuclear translocation of β-catenin, maintaining expression of this protein at the cell membrane, which leads to increased cell-cell adhesion and inhibition of the WNT pathway [30]. These NDRG1-mediated activities further contribute to decreasing cancer cell migration. In fact, NDRG1 plays a significant role in reducing cancer cell migration by inhibiting the Rho-associated coiled-coil containing protein kinase1 (ROCK1)/phosphorylated myosin light chain2 (pMLC2) pathway, which is downstream of the Rho family of small GTPases, to regulate F-actin polymerization and organization [31]. However, the mechanisms by which NDRG1 mediates its effects on cancer cell migration were not fully elucidated and require further investigation.

These previous studies have led to the current investigation, which examined the effect of NDRG1 on the activation of c-Src, as well as its downstream effectors, p130Cas and c-Abl, in terms of regulating a critical modulator of cell migration, Rac1. Herein, for the first time, our investigations demonstrated that NDRG1 inhibits c-Src activation by down-regulating EGFR expression and attenuating EGF-induced EGFR activation, leading to a reduction in EGFR-c-Src interactions. NDRG1 suppressed Rac1 activity through c-Src-dependent down-regulation of p130Cas signaling, and thus, suppressed the ability of Rac1 to promote cell migration. Moreover, NDRG1 also inhibited the c-Abl-CrkII pathway by a c-Src-independent mechanism. Finally, novel and potent compounds that up-regulate NDRG1 and are currently under development as anti-metastatic agents, markedly decreased c-Src activation. These studies are critical for understanding the potent role of NDRG1 in preventing cancer metastasis and how to target these important pathways with therapeutics in the future.

## RESULTS

### NDRG1 suppresses the activation of c-Src

Many proto-oncogenes regulate cell signaling involved in migration, with c-Src being critical in modulating these pathways [3]. However, the effect of NDRG1 on c-Src activation and its downstream targets (Figure 1A) have not been elucidated and were the subject of this investigation. Initially, to elucidate the molecular role of NDRG1 on regulating the activation of c-Src, we utilized two established models, namely DU145 prostate cancer cells (Figure 1B) and HT29 colon cancer cells (Figure 1C) that stably over-express exogenous human NDRG1 (denoted as “NDRG1”). These cells were implemented herein as we have previously shown that NDRG1 expression decreases cell migration and invasion in these two cell-types [31]. In these two cell lines, a ~45 kDa band was detected by immunoblots and represents exogenous expression of FLAG-tagged NDRG1 (Figure 1B, 1C). Furthermore, endogenously expressed NDRG1 (*e.g*., in vector control cells) was detected at ~43 and 44 kDa, suggesting potential post-translational processing of this protein [32, 33]. Regarding these observations, the densitometric assessment of NDRG1 in immunoblots throughout this study represents the total of all NDRG1 bands. The NDRG1-transfected DU145 (Figure 1B) and HT29 cells (Figure 1C) showed a significant (*p* < 0.001) increase in NDRG1 expression compared to their empty vector-transfected control (vector control) cells.

As additional models to investigate the effects of endogenous NDRG1, NDRG1-silenced clones (sh-NDRG1) of these two cell-types were generated [29]. As indicated in Figure 1B, 1C, compared to control cells transfected with scrambled shRNA (sh-control), the sh-NDRG1 clones demonstrated a significant (*p* < 0.01) decrease in NDRG1 levels in DU145 (Figure 1B) and HT29 cells (Figure 1C). Considering different cancers possess markedly altered genetic backgrounds, all of our current experiments were performed with at least both cell-types in order to assess NDRG1 function. In fact, in several studies, PC3MM prostate cancer cells were also assessed (Supplementary Figure 1A, Supplementary Figure 3A–3B), which demonstrated similar results to both DU145 and HT29 cells.

To determine the role of the c-Src tyrosine kinase in NDRG1-mediated inhibition of cell migration, the expression and phosphorylation of c-Src were analyzed. Considering c-Src activation, auto-phosphorylation of this protein at Tyr416 in the kinase domain plays a critical role in increasing c-Src tyrosine kinase activity [34]. In contrast, phosphorylation at Tyr527 leads to an inhibitory effect on c-Src activity [35]. Therefore, the phosphorylation status of these sites was initially examined to decipher if NDRG1 could affect c-Src activation. As shown in Figure 1B, 1C for both DU145 and HT29 cells, NDRG1 over-expression significantly (*p* < 0.01) decreased c-Src phosphorylation at Tyr416 relative to that observed in vector control cells, while it had no significant (*p* > 0.05) effect on c-Src phosphorylation at Tyr527 or total c-Src levels. Studies using a third cancer cell-type, namely PC3MM prostate cancer cells, also showed similar results to those observed using DU145 and HT29 cells (Supplementary Figure 1A).

In contrast, NDRG1 silencing in DU145 and HT29 cells significantly (*p* < 0.001) increased c-Src phosphorylation levels at Tyr416 relative to their corresponding sh-control cells, without significantly (*p* > 0.05) affecting phosphorylation of c-Src at Tyr527 *versus* sh-control cells (Figure 1B, 1C). Interestingly, in DU145 cells, while NDRG1 over-expression did not significantly (*p* > 0.05) alter total c-Src levels, silencing of NDRG1 led to a slight, but significant (*p* < 0.05) increase in total c-Src relative to the sh-control cells (Figure 1B). However, a different response was observed in HT29 cells, where NDRG1 silencing did not significantly (*p* > 0.05) affect total c-Src protein relative to sh-control cells (Figure 1C). This difference in response may be related to the diverse molecular backgrounds of these two cell-types.

Collectively, these studies demonstrated that NDRG1 over-expression and silencing reduced and elevated c-Src phosphorylation at Tyr416, respectively, which indicates, for the first time, that NDRG1 expression regulates c-Src activity.

### NDRG1 reduces EGFR expression and abrogates EGF-induced EGFR activation, subsequently inhibiting the EGFR-c-Src association and c-Src activation

The above studies suggest that NDRG1 has a unique role in modulating the activity of c-Src in prostate and colon cancer cells, which is an important proto-oncogene integrally involved in cell transformation, proliferation, angiogenesis and migration [36]. To dissect the molecular mechanisms underlying the effect of NDRG1 in altering c-Src signaling, several key regulators of its activation and kinase activity were analyzed, including the receptor tyrosine kinases, EGFR and PDGFR, as well as the c-Src-modulating phosphotyrosine phosphatases, PTP1B and PTP-PEST [3, 5, 37–39].

**Figure 2:**
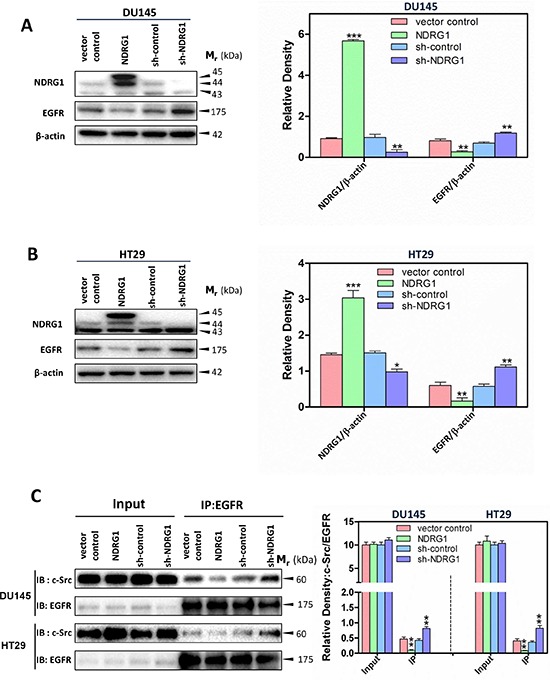
NDRG1 decreased EGFR expression and c-Src binding to EGFR Immunoblotting was conducted to examine NDRG1 and EGFR expression, using both NDRG1 over-expressing and silencing models in **(A)** DU145 and **(B)** HT29 cells. Immunoblot analysis was performed as described in the legend for Figure 1 and demonstrated that NDRG1 decreased the expression of EGFR in DU145 and HT29 cells. **(C)** Co-immunoprecipitation to assess the binding of EGFR with c-Src was performed as described in the *Materials and Methods* using an EGFR antibody for co-immunoprecipitation and c-Src or EGFR antibodies for immunoblotting. These studies showed that NDRG1 expression prevented c-Src binding to EGFR, which inhibited the stimulatory effect of EGFR on c-Src activation. Immunoblots shown are representative of three independent experiments. Densitometry data are mean ± S.D. (3–5 experiments); *p < 0.05; **p < 0.01; ***p < 0.001, relative to vector control or sh-control cells, as appropriate.

It has been demonstrated that EGFR is frequently deregulated in human tumors, such as prostate and colon tumors, triggering downstream oncogenic signaling pathways [40]. The over-expression of EGFR and/or EGF-binding to EGFR can result in its auto-phosphorylation, leading to its coupling to c-Src [41, 42]. This event activates c-Src auto-phosphorylation of Tyr416 by disrupting its intra-molecular closed conformation [41, 42]. Given the known role of EGFR in promoting c-Src activity, we initially examined whether NDRG1 plays a role in EGFR expression. Interestingly, immunoblot analysis revealed a significant (*p* < 0.01) decrease in total EGFR levels in response to NDRG1 over-expression in DU145 and HT29 cells compared to the respective vector controls (Figure 2A, 2B). In contrast, compared with sh-control cells, silencing NDRG1 led to significantly (*p* < 0.01) increased EGFR expression in DU145 and HT29 cells (Figure 2A, 2B). These observations suggest NDRG1 decreases EGFR expression, which could prevent its stimulatory effect on c-Src, accounting for the suppression of c-Src activation upon NDRG1 expression (Figure 1B, 1C).

To further assess the role of EGFR in altering c-Src activation following changes in NDRG1 expression, the above studies were complemented by incubating these cells with or without the EGF ligand, which activates EGFR auto-phosphorylation and triggers its downstream oncogenic signaling cascades [43]. Immunoblotting data showed that for both cell-types, NDRG1 expression was not significantly (*p* > 0.05) affected by EGF treatment under all conditions (Supplementary Figure 1B, 1C). However, incubation of vector control and sh-control DU145 or HT29 cells with EGF led to a pronounced and significant (*p* < 0.01) increase in phosphorylated EGFR at Tyr1148, relative to that observed in untreated cells (Supplementary Figure 1B, 1C). In contrast, NDRG1 over-expression markedly and significantly (*p* < 0.01) diminished the ability of EGF to elevate EGFR phosphorylation (Tyr1148; Supplementary Figure 1B, 1C). When assessing the effect of EGF in DU145 and HT29 sh-NDRG1 models, the level of EGFR phosphorylation (Tyr1148) was significantly (*p* < 0.01) promoted by EGF in sh-NDRG1 models relative to sh-control cells (Supplementary Figure 1B, 1C). Hence, this observation indicated an inhibitory effect of NDRG1 on EGF-induced EGFR phosphorylation at Tyr1148, which is important for stimulating c-Src activation [42].

As shown in Figure 2A, 2B for DU145 and HT29 cells in the absence of EGF, NDRG1 over-expression also significantly (*p* < 0.01) decreased total EGFR protein levels *versus* vector control cells, while sh-NDRG1 resulted in a significant (*p* < 0.01–0.05) increase in EGFR relative to the sh-control (Supplementary Figure 1B, 1C). In contrast to the distinct and similar effect of EGF on the phosphorylation of EGFR in both DU145 and HT29 cells, its effect on total EGFR was not as marked or as consistent in these cell-types (Supplementary Figure 1B, 1C). In terms of DU145 cells, EGF did not have any significant (*p* > 0.05) effect on EGFR levels in the vector control or sh-control cells relative to these cells without incubation with EGF (Supplementary Figure 1B). Conversely, EGF significantly (*p* < 0.05) decreased EGFR in NDRG1 over-expressing and sh-NDRG1 DU145 cells *versus* these cells without EGF (Supplementary Figure 1B). Using HT29 cells, EGF significantly (*p* < 0.01) decreased EGFR levels in vector control and sh-NDRG1 cells relative to these cells treated without EGF, while having no significant (*p* > 0.05) effect on EGFR levels in NDRG1 over-expressing or sh-control cells (Supplementary Figure 1C).

Importantly, EGF treatment of DU145 or HT29 cells led to a similar general effect on c-Src phosphorylation at Tyr416 as that obtained examining EGFR phosphorylation at Tyr1148 (Supplementary Figure 1B, 1C). That is, NDRG1 over-expression significantly (*p* < 0.01–0.05) decreased the effect of EGF on phosphorylated c-Src (Tyr416) levels relative to the vector control, while NDRG1 silencing significantly (*p* < 0.05) enhanced this effect relative to the sh-control. Incubation with EGF had no significant (*p* > 0.05) effect on total c-Src levels when compared to each respective control without EGF under all conditions in both cell-types (Supplementary Figure 1B, 1C). However, as demonstrated in Figure 1B, silencing NDRG1 significantly (*p* < 0.05) up-regulated total c-Src levels in DU145 cells only (Supplementary Figure 1B). Collectively, these data revealed that NDRG1 over-expression abrogates EGF-induced EGFR activation and attenuates its effect on c-Src activation. These observations suggest regulation of EGFR expression and inhibition of its activation by NDRG1 could be responsible for the ability of this metastasis suppressor to inhibit c-Src kinase activation.

### NDRG1 decreases EGFR-binding to c-Src

To further examine the mechanism of the NDRG1 mediated down-regulation of c-Src activation *via* EGFR, immunoprecipitation experiments were performed to assess the effect of NDRG1 on the EGFR and c-Src protein-protein interaction in DU145 and HT29 cells (Figure 2C). Interestingly, NDRG1 over-expression significantly (*p* < 0.01) reduced c-Src binding to EGFR relative to the vector control, whereas sh-NDRG1 significantly (*p* < 0.01) promoted the association of c-Src with EGFR relative to the sh-control in both cell-types (Figure 2C). In summary, the immunoprecipitation results, when taken together with the immunoblotting data, suggested NDRG1 affected c-Src activation *via* decreasing EGFR expression, leading to loss of activated EGFR, and thus, preventing the EGFR-c-Src interaction.

### Effects of NDRG1 on other regulators of c-Src activation

Dephosphorylation of c-Src at p-Tyr527 by phosphotyrosine phosphatases (*e.g*., PTP1B and PTP-PEST) plays an important role in regulating the activity of this kinase [5]. To gain further insight into the regulation of c-Src activation upon NDRG1 expression, we also assessed PTP1B and PTP-PEST levels, which are the most characterized among this group of phosphatases [6, 44]. However, there was no consistent effect on the expression of these phosphatases after NDRG1 over-expression or silencing in both cell-types (Supplementary Figure 2A, 2B).

Another receptor tyrosine kinase that is known to activate c-Src, namely PDGFR [39], was also examined upon incubation with PDGF in both cell-types in response to NDRG1. However, we found that NDRG1 had no marked or consistent effect on this latter molecule or its activation after incubation with PDGF (data not shown). Therefore, these observations suggest that the regulation of c-Src activation by NDRG1 mainly occurred through EGFR.

### NDRG1 inhibits p130Cas phosphorylation and consequently the binding of p130Cas to CrkII

The studies above demonstrated that NDRG1 has a novel role in regulating c-Src activation through its ability to down-regulate EGFR levels and activation, indicating that NDRG1 may exert, at least in part, its anti-metastasis function by inhibiting the oncogenic effect of c-Src. It has been reported that c-Src could interact with two molecules, namely p130Cas and c-Abl, to modulate the signaling cascades involved in c-Src-induced cancer cell migration (Figure 1A) [13, 14]. Phosphorylation of p130Cas by c-Src is known to activate this protein, which subsequently recruits unphosphorylated CrkII to form a protein-protein complex containing the guanine nucleotide exchange factor, DOCK180, that acts as a “molecular switch” to activate Rac1 [45]. This event results in the modulation of cytoskeleton dynamics and leads to the formation of filopodia, lamellipodia, membrane ruffles and ultimately cell migration [13, 45].

**Figure 3:**
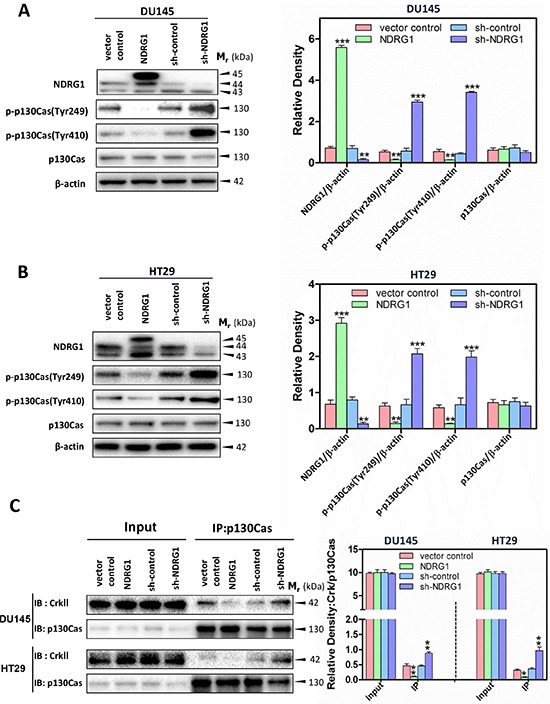
NDRG1 expression decreased p130Cas phosphorylation and subsequently the binding of p130Cas and CrkII in DU145 and HT29 cells **(A, B)** Immunoblotting revealed that NDRG1 expression reduced p130Cas phosphorylation at Tyr249 and Tyr410 in both DU145 (A) and HT29 (B) cells. **(C)** Co-immunoprecipitation demonstrated that, for both DU145 and HT29 cells, NDRG1 expression decreased the binding of p130Cas and CrkII. Immunoblots shown are representative of three independent experiments. Densitometry data are mean ± S.D. (3–5 experiments); *p < 0.05; **p < 0.01; ***p < 0.001, relative to vector control or sh-control cells, as appropriate.

Considering the inhibitory effect of NDRG1 expression on c-Src activation (Figure 1B, 1C), it was important to examine whether NDRG1 could inhibit signaling targets downstream of c-Src to decrease tumor cell migration. Studies were first conducted to investigate the levels and phosphorylation of p130Cas upon NDRG1 over-expression and silencing (Figure 3A, 3B). For both DU145 and HT29 cells, over-expression of NDRG1 markedly and significantly (*p* < 0.01) reduced the phosphorylation of p130Cas at Tyr249 and Tyr410, relative to vector control cells (Figure 3A, 3B). Notably, the phosphorylation of residues Tyr249 and Tyr410, which are located in the substrate-binding domain, are important for p130Cas activation [11]. Thus, NDRG1 overexpression decreased p130Cas activation. A similar effect of NDRG1 over-expression on significantly (*p* < 0.05) suppressing the phosphorylation of p130Cas at Tyr249 and Tyr410 was also observed in PC3MM cells (Supplementary Figure 3A). In contrast, NDRG1 silencing caused a pronounced and significant (*p* < 0.001) increase in phosphorylated p130Cas at Tyr249 and Tyr410 in DU145 and HT29 cells relative to sh-controls (Figure 3A, 3B). On the other hand, despite the impact of NDRG1 on p130Cas phosphorylation, there was no significant (*p* > 0.05) change in total p130Cas levels in either NDRG1 over-expressing or silenced DU145 and HT29 cells (Figure 3A, 3B). These data demonstrate that NDRG1 expression inhibited the activation of p130Cas, which is a key substrate and downstream effector of c-Src involved in signaling pathways contributing to cellular migration [45].

Given that phosphorylation of p130Cas promotes its binding to unphosphorylated CrkII, which then activates Rac1 [13], immunoprecipitation studies were then performed to further establish whether NDRG1 plays a role in the interaction of p130Cas and CrkII (Figure 3C). In these experiments, NDRG1 over-expression significantly (*p* < 0.01–0.05) decreased the p130Cas and CrkII association in both DU145 and HT29 cells relative to the vector controls, while silencing NDRG1 led to a significant (*p* < 0.01) increase in this interaction relative to sh-control cells (Figure 3C). These results strongly support the hypothesis that NDRG1 decreases the effect of c-Src oncogenic activation, leading to reduced p130Cas phosphorylation, as well as binding of p130Cas to CrkII, which may inhibit Rac1 activation.

### NDRG1 inhibits the activity of Rac1 and its downstream target by modulating c-Src activation

**Figure 4:**
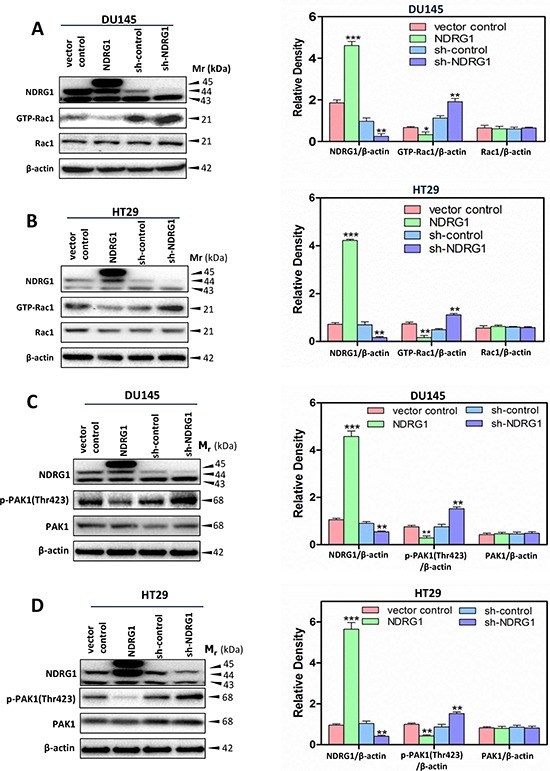
NDRG1 suppressed Rac1 activity and its downstream effector PAK1 **(A, B)** A Rac1 activation assay was performed (see *Materials and Methods*) to detect the active form of Rac1 (GTP-Rac1) in (A) DU145 and (B) HT29 cells. These studies demonstrated that NDRG1 expression inhibited Rac1 activity. **(C, D)** Immunoblotting showed that NDRG1 suppressed PAK1 phosphorylation (Thr423) in (C) DU145 and (D) HT29 cells. Immunoblotting results are representative of three independent experiments. Densitometry data are mean ± S.D. (3–5 experiments); *p < 0.05; **p < 0.01; ***p < 0.001, relative to vector control or sh-control cells, as appropriate.

To further investigate the finding that NDRG1 plays a negative regulatory role in c-Src activation and its downstream effectors (namely p130Cas; Figures 1, 3), a Rac1 activation assay was performed to assess the effect of NDRG1 on Rac1 activity. This was examined as there is a close association between c-Src and Rac1 activity linked by the p130Cas-CrkII-DOCK180 complex [46]. For both DU145 and HT29 cells, NDRG1 over-expression significantly (*p* < 0.01–0.05) inhibited Rac1 activation, which was determined by the levels of GTP-bound Rac1 (GTP-Rac1), relative to vector control cells (Figure 4A, 4B). In contrast, silencing NDRG1 significantly (*p* < 0.01) increased GTP-Rac1 levels relative to the sh-control DU145 and HT29 cells (Figure 4A, 4B). Although alteration in NDRG1 expression markedly influenced GTP-Rac1 levels, it did not significantly (*p* > 0.05) affect total Rac1 levels in both cell-types (Figure 4A, 4B).

Further investigations were then conducted to expand our understanding of NDRG1 on Rac1 activity by examining phosphorylation of p21 activated kinase 1 (PAK1), which is a direct downstream signaling target of activated Rac1 (Figure 4C, 4D) [47]. Rac1 activation results in PAK1 phosphorylation, which leads to cytoskeletal remodeling, alterations in cell adhesion, as well as the EMT, all of which are required for promoting cancer cell migration [48]. Interestingly, NDRG1 over-expression significantly (*p* < 0.01) decreased PAK1 phosphorylation at Thr423 without any significant (*p* > 0.05) effect on total PAK1 levels relative to vector control cells in both cell-types (Figure 4C, 4D). Conversely, silencing NDRG1 in DU145 and HT29 cells led to a significant (*p* < 0.01) increase of phosphorylated PAK1 at Thr423 relative to sh-control cells, while there was no significant (*p* > 0.05) alteration in total PAK1 levels (Figure 4C, 4D). Hence, these observations further confirmed that NDRG1 expression leads to suppressed Rac1 activity (Figure 4).

**Figure 5:**
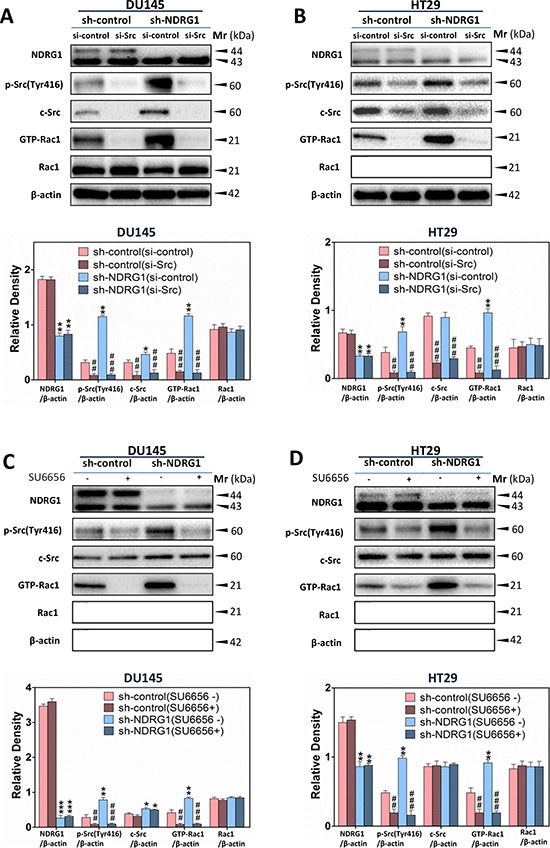
NDRG1 inhibited Rac1 activation in a c-Src-dependent manner **(A, B)** Immunoblotting revealed that, for (A) DU145 and (B) HT29 cells, c-Src silencing by specific siRNA significantly reduced Rac1 activity relative to the si-control. **(C, D)** Using immunoblotting, Rac1 activity was shown to be significantly decreased by SU6656, an established c-Src inhibitor in (C) DU145 and (D) HT29 cells. Immunoblotting results are representative of three independent experiments. Densitometry data are mean ± S.D. (3–5 experiments); *p < 0.05; **p < 0.01; ***p < 0.001, relative to sh-control (si-control) cells, ^#^*p* < 0.05; ^##^*p* < 0.01; ^###^*p* < 0.001, relative to c-Src si-control cells (A, B) or cells incubated with control medium only (C, D), as appropriate.

Next, we sought to examine whether NDRG1-induced inhibition of Rac1 activity was dependent on the regulation of c-Src activation. Additional studies were implemented to silence c-Src using c-Src siRNA (Figure 5A, 5B), or by pharmacologically inhibiting c-Src signaling using a well characterized c-Src kinase inhibitor (SU6656 [18]; Figure 5C, 5D), in both DU145 and HT29 sh-control and sh-NDRG1 cells. We initially incubated NDRG1-silenced cell models and their respective sh-control cells in both cell-types with c-Src-specific siRNA (si-Src) to transiently silence endogenous c-Src expression relative to the negative si-control (Figure 5A, 5B).

As expected, si-Src had no significant (*p* > 0.05) effect on NDRG1 expression relative to the si-control-treated cells in both cell-types (Figure 5A, 5B). Notably, phosphorylated c-Src (Tyr416) and total c-Src were significantly (*p* < 0.001–0.01) reduced in both DU145 and HT29 cells upon treatment with si-Src compared to the si-control under both the sh-control and sh-NDRG1 conditions (Figure 5A, 5B). Silencing c-Src also resulted in a significant (*p* < 0.001–0.01) decrease in the activated form of Rac1 relative to the si-controls. In contrast, c-Src silencing did not significantly (*p* > 0.05) alter total levels of Rac1 in sh-control and sh-NDRG1 cells relative to the si-control in DU145 and HT29 cells (Figure 5A, 5B).

When using the pharmacological c-Src inhibitor, SU6656 [18], there was no significant (*p* > 0.05) alteration in NDRG1 levels in these cell models (Figure 5C, 5D). Therefore, this observation excluded the possibility of a non-specific effect of the inhibitor on NDRG1 expression. On the other hand, SU6656 significantly (*p* < 0.001–0.01) down-regulated the phosphorylation of c-Src at Tyr416 relative to the control, but had no significant (*p* > 0.05) effect on total c-Src levels (Figure 5C, 5D). Furthermore, SU6656 significantly (*p* < 0.001–0.01) decreased activated Rac1, without significantly (*p* > 0.05) affecting total Rac1 in both DU145 and HT29 sh-control and sh-NDRG1 cells (Figure 5C, 5D). Hence, collectively from these studies using c-Src siRNA and SU6656, it can be concluded that NDRG1 decreases Rac1 activity in a c-Src-dependent manner.

Taken together, the data presented in Figures 1–5 and Supplementary Figures 1–3 demonstrated that NDRG1 inhibits c-Src activation through abrogation of the EGFR and c-Src interaction. This effect leads to reduced c-Src activation and decreased p130Cas phosphorylation and activation, which inhibits p130Cas-binding to CrkII, and hence, decreases Rac1 activity and PAK1 activation.

### NDRG1 inhibits the c-Abl-CrkII pathway

**Figure 6:**
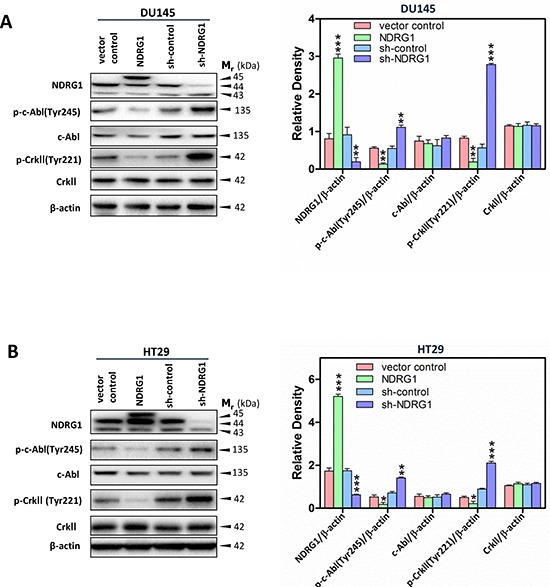
NDRG1 expression inhibited c-Abl activation and its effect on CrkII phosphorylation **(A, B)** Immunoblot analysis demonstrated that NDRG1 decreased c-Abl phosphorylation at Tyr245 and CrkII phosphorylation at Tyr221, while having no significant effect on total c-Abl and CrkII expression in DU145 (A) and HT29 (B) cells. Immunoblotting results in (A, B) are representative of three experiments. Densitometry data are mean ± S.D. (3–5 experiments); *p < 0.05; **p < 0.01; ***p < 0.001, relative to vector control or sh-control cells, as appropriate.

To further establish the role of NDRG1 on modulating downstream effectors of c-Src, studies were conducted to examine the effect of altering NDRG1 expression on c-Abl phosphorylation, which leads to its activation that subsequently stimulates cell migration [49, 50]. Over-expression of NDRG1 in DU145 and HT29 cells significantly (*p* < 0.01–0.05) decreased c-Abl phosphorylation at Tyr245 (Figure 6A, 6B), while it showed no significant (*p* > 0.05) effect on total c-Abl levels relative to vector control cells. This effect was also observed in PC3MM cells, where NDRG1 expression significantly (*p* < 0.01) inhibited phosphorylation of c-Abl at Tyr245, again, with no marked alteration in total c-Abl levels relative to control cells (Supplementary Figure 3B). In contrast, silencing NDRG1 caused a pronounced (*p* < 0.01) increase in phosphorylated c-Abl at Tyr245 and similarly demonstrated no significant (*p* > 0.05) effect on the total level of c-Abl in both DU145 and HT29 cells relative to sh-control cells (Figure 6A, 6B). Taken together, these data support a role for NDRG1 in inhibiting c-Abl activation in both the prostate cancer cell lines, DU145 and PC3MM, and the colon cancer cell line, HT29.

Previous studies have established that c-Abl can regulate cell migration and cell adhesion *via* modulating the CrkII-Rac1 signaling pathway [16, 51, 52] (Figure 1A). In fact, c-Abl is a major regulator of CrkII activation, which regulates CrkII *via* a phosphorylation at Tyr221 [53]. While phosphorylation at Tyr221 may prevent binding of CrkII to p130Cas [53], it has been shown to be responsible for the activation of the Rac1 signaling pathway and cell migration, possibly *via* an alternative signaling pathway [15, 16] (Figure 1A).

Therefore, we assessed CrkII phosphorylation at Tyr221 following alterations in NDRG1 expression, in order to further establish the mechanisms underlying NDRG1-mediated-suppression of cancer cell migration [29, 31, 54]. Upon NDRG1 over-expression, CrkII phosphorylation at Tyr221 was significantly (*p* < 0.01–0.05) decreased relative to vector control in DU145, HT29 and PC3MM cells, while NDRG1 led to no significant (*p* > 0.05) alteration in total CrkII expression (Figure 6A, 6B; Supplementary Figure 3B). On the other hand, silencing NDRG1 induced a significant (*p* < 0.001) increase in the phosphorylation of CrkII at Tyr221 in both DU145 and HT29 cells relative to sh-control cells (Figure 6A, 6B). However, silencing of NDRG1 did not significantly (*p* > 0.05) alter the total CrkII levels relative to the sh-control in both cell-types (Figure 6A, 6B). Hence, collectively, these studies demonstrate that NDRG1 also inhibits the c-Abl-CrkII pathway.

### c-Src siRNA and the c-Src kinase inhibitor, SU6656, demonstrate that the NDRG1-induced reduction of p130Cas is due to the effect of this metastasis suppressor on c-Src

**Figure 7:**
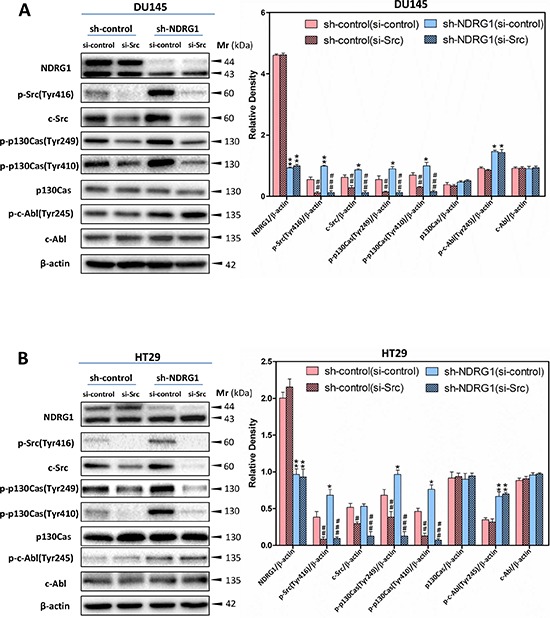
c-Src-specific siRNA inhibited c-Src-induced p130Cas phosphorylation at Tyr249 and Tyr410, but not c-Abl phosphorylation at Tyr245 **(A, B)** Immunoblotting demonstrating that the silencing of c-Src results in a significant reduction in phosphorylated p130Cas (Tyr249 and Tyr410) levels for both DU145 (A) and HT29 (B) cells, while having no significant effect on c-Abl phosphorylation (Tyr245). Immunoblotting is representative of three experiments. Densitometry data are mean ± S.D. (3–5 experiments); *p < 0.05; **p < 0.01; ***p < 0.001, relative to sh-control (si-control) cells, *^#^p* < 0.05; ^##^*p* < 0.01; ^###^*p* < 0.001, relative to sh-control (si-control) or sh-NDRG1 (si-control) cells, as appropriate.

**Figure 8:**
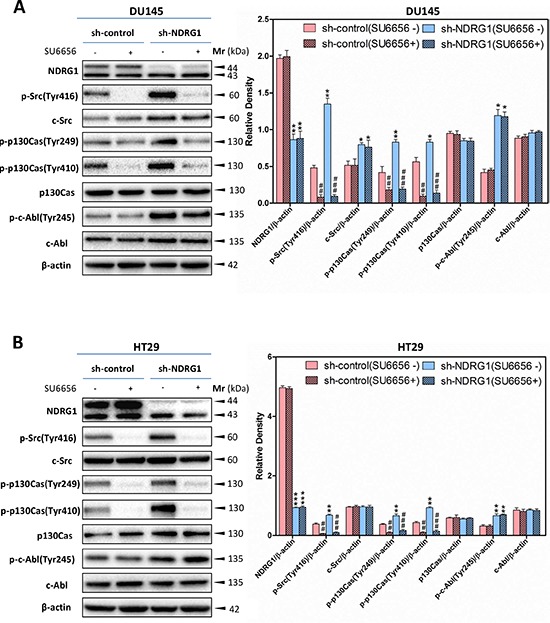
The c-Src inhibitor, SU6656, decreased the level of phosphorylated p130Cas at Tyr249 and Tyr410, but not c-Abl phosphorylation at Tyr245 **(A, B)** SU6656 inhibition of c-Src catalytic activity led to significantly decreased p130Cas phosphorylation at Tyr249 and Tyr410 in both DU145 (A) and HT29 (B) sh-control and sh-NDRG1 cells. In contrast, SU6656 incubation does not affect c-Abl phosphorylation (Tyr245). Immunoblotting results are representative of three experiments. Densitometry data are mean ± S.D. (3–5 experiments); *p < 0.05; **p < 0.01; ***p < 0.001, relative to sh-control cells, *^#^p* < 0.05; ^##^*p* < 0.01; *^###^p* < 0.001, relative to sh-control or sh-NDRG1 cells incubated with control medium only, as appropriate.

To examine whether the effect of NDRG1 on p130Cas and c-Abl phosphorylation is dependent on the regulation of c-Src activation, investigations were implemented using c-Src siRNA (Figure 7A, 7B), or the c-Src kinase inhibitor, SU6656 [18] (Figure 8A, 8B), to silence c-Src or pharmacologically inhibit c-Src activity, respectively, in DU145 and HT29 sh-control and NDRG1-silenced cells.

As also shown in Figure 5A and 5B, *p*-Src (Tyr416) and total c-Src were significantly (*p* < 0.001–0.05) reduced in both DU145 and HT29 cells upon treatment with si-Src compared to the si-controls under both the sh-control and sh-NDRG1 conditions (Figure 7A, 7B). Importantly, the silencing of c-Src was accompanied by a significant (*p* < 0.001–0.01) reduction in p130Cas phosphorylation at Tyr249 and Tyr410 regardless of NDRG1 expression in both cell-types, while it had no significant (*p* > 0.05) effect on total p130Cas protein levels in both sh-control and sh-NDRG1 cells (Figure 7A, 7B). These data clearly demonstrate that the modulation of p130Cas activation by NDRG1 occurs in a c-Src-dependent manner. However, for both cell-types, silencing c-Src did not alter either phosphorylated c-Abl (Tyr245) or total c-Abl. This suggested that c-Abl was not a direct target for c-Src under these conditions and NDRG1-mediated regulation of c-Abl activation occurred through a c-Src independent mechanism.

The observations above were complemented by incubating NDRG1 sh-control and silenced DU145 and HT29 cells with the c-Src inhibitor, SU6656 [18] (Figure 8A, 8B). Indeed, after incubation of sh-control or sh-NDRG1 DU145 or HT29 cells with SU6656 (10 μM) for 1 h/37°C, c-Src phosphorylation at Tyr416 was significantly (*p* < 0.001–0.01) reduced relative to the controls (Figure 8A, 8B). In contrast, under these conditions, SU6656 did not significantly (*p* > 0.05) affect total c-Src protein levels relative to the controls. Importantly, for both sh-control and sh-NDRG1 cells, the inhibitory effect of SU6656 on c-Src was repeated in terms of its effect on phosphorylated p130Cas, where a significant (*p* < 0.001–0.01) decrease in phosphorylation of p130Cas at Tyr249 and Tyr410 was observed when compared to cells incubated with the control (Figure 8A, 8B). There was no significant (*p* > 0.05) effect of SU6656 on total p130Cas levels relative to the control in both cell-types. Moreover, relative to the control, there was no significant (*p* > 0.05) change in c-Abl phosphorylation or the total amount c-Abl upon treatment with SU6656 in DU145 or HT29 cells. Together, our results demonstrated that SU6656 inhibited c-Src activity (Figure 8A, 8B), and in good agreement with the c-Src siRNA studies (Figure 7A, 7B), provided further evidence that NDRG1 modulates p130Cas activation, but not c-Abl, by inhibition of c-Src activity.

### NDRG1 reduces cancer cell migration through modulating c-Src activation

We previously discovered that NDRG1 plays a novel role in decreasing cancer cell migration by targeting the TGF-β-mediated EMT [29] and ROCK/pMLC2 pathways [31]. Based on the current study, an intriguing hypothesis was that down-regulation of c-Src by NDRG1 was also engaged in this anti-oncogenic effect of suppressing tumor cell migration. Hence, we conducted migration assay experiments to assess the role played by c-Src in modulating cell migration and whether the reduction of c-Src activation in response to NDRG1 expression can decrease migration. Again, these studies were performed using c-Src siRNA and SU6656 in DU145 and HT29 sh-control and NDRG1-silenced cells. Cell migration was examined through a modified Boyden chamber using xCELLigence real-time cell analysis that is based on impedance-based detection of migrating cells [54], to assess the effect of NDRG1.

**Figure 9:**
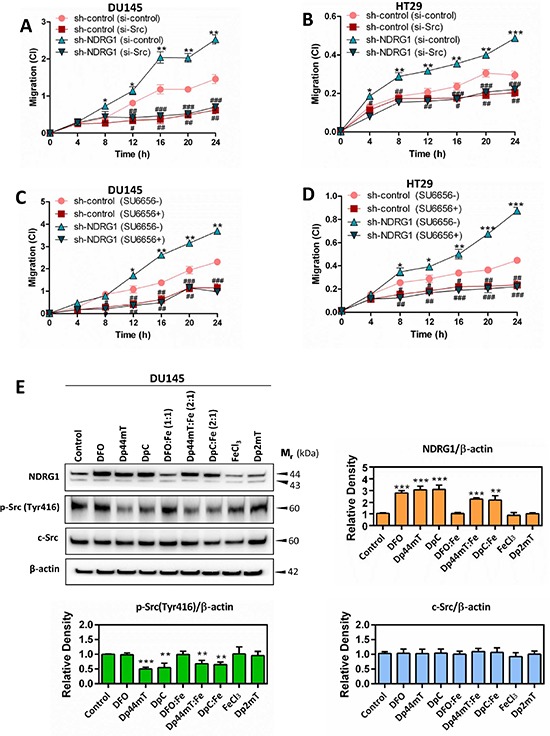
NDRG1 decreases cancer cell migration in a c-Src-dependent manner (A–D) Dp44mT and DpC increase NDRG1 expression and also decrease activation of c-Src (E) (A–D) Migration assays demonstrating that NDRG1 silencing significantly increased DU145 (A, C) and HT29 (B, D) cell migration, as determined by the xCELLigence real-time cell analysis migration assay (see *Materials and Methods*). In contrast, transiently silencing of c-Src with siRNA, or inhibition of c-Src activity with SU6656, reversed the effect of silencing NDRG1. (E) The levels of NDRG1, p-Src(Tyr416) and c-Src measured by western analysis in DU145 following a 24 h/37°C incubation with control medium, DFO (250 μM), Dp44mT (5 μM), DpC (5 μM), DFO:Fe (1:1; 250 μM), Dp44mT:Fe (2:1; 5 μM), DpC:Fe (2:1; 5 μM), FeCl_3_ (250 μM), or Dp2mT (5 μM). Results are expressed as mean ± S.D. (3–5 experiments); *p < 0.05; **p < 0.01; ***p < 0.001, relative to sh-control cells, ^#^*p* < 0.05; ^##^*p* < 0.01; ^###^*p* < 0.001, relative to sh-control (si-control) or sh-NDRG1 (si-control) cells, as appropriate (A, B); or *p < 0.05; **p < 0.01; ***p < 0.001, relative to sh-control cells, ^#^*p* < 0.05; ^##^*p* < 0.01; ^###^*p* < 0.001, relative to sh-control or sh-NDRG1 cells incubated with control medium only, as appropriate (C, D); *p < 0.05; **p < 0.01; ***p < 0.001, relative to cells incubated with control medium only (E).

As demonstrated in Figure 9A–9D, relative to DU145 or HT29 sh-control cells (si-control), there was a significant (*p* < 0.05) increase in the migratory capacity of sh-NDRG1 cells (si-control) after only 4–8 h of incubation and remained significantly (*p* < 0.001–0.05) increased for up to 24 h. This is consistent with our recent reports demonstrating that inhibition of NDRG1 expression increases cellular migration [29, 31]. However, upon silencing c-Src, a significant (*p* < 0.001–0.05) decrease in migration was observed in both sh-control and sh-NDRG1 cells in comparison to si-control transfected cells for both cell-types (Figure 9A, 9B). Importantly, these observations were confirmed by incubating cells with the c-Src inhibitor, SU6656, upon which the migration of sh-control and sh-NDRG1 cells were significantly (*p* < 0.001–0.05) reduced in both cell-types relative to the control (Figure 9C, 9D). In summary, these observations above indicate that silencing NDRG1 increases cell migration relative to the control and that inhibition of c-Src can prevent this increase. Hence, the role of NDRG1 in decreasing cell migration is, at least in part, due to its effects on inhibiting c-Src activation.

### The potent NDRG1-inducing agents, DpC and Dp44mT, markedly decrease phosphorylation of c-Src at Tyr416

The studies above indicate that NDRG1 markedly suppresses c-Src activation and its subsequent downstream pathways to result in inhibition of cellular migration that is crucial for metastasis. Hence, targeting this pathway could lead to a critical new therapeutic strategy. Interestingly, thiosemicarbazones of the di-2-pyridylketone thiosemicarbazone (DpT) class, including di-2-pyridylketone 4, 4, -dimethyl-3-thiosemicarbazone (Dp44mT) and di-2-pyridylketone 4-cyclohexyl-4-methyl-3-thiosemicarbazone (DpC), have been demonstrated to act as effective agents to induce NDRG1 expression [55, 56]. These compounds markedly inhibit tumor cell proliferation and migration *in vitro* [29, 31], as well as tumor growth and metastasis *in vivo* [28, 56–59]. The mechanism of action of the DpT class of compounds occurs by binding cellular iron, leading to hypoxia inducible factor-1α (HIF-1α)-dependent and -independent mechanisms, which then increase *NDRG1* transcription [55]. Furthermore, the action of these agents also involves the formation of redox active metal complexes, such as their iron complexes (*i.e*., Dp44mT:Fe or DpC:Fe), which then result in the generation of cytotoxic reactive oxygen species (ROS) [57, 59].

To assess the effect of Dp44mT and DpC on c-Src activation, their efficacy were compared to the negative control compound, di-2-pyridylketone 2-methyl-3-thiosemicarbazone (Dp2mT), which is a structural analog that cannot bind metals [29, 57, 60] (Figure 9E). Further, their effects were compared to the drug, desferrioxamine (DFO), which binds cellular iron, but does not redox cycle to generate ROS [61, 62], and also FeCl_3_ alone, which was used to synthesize the Dp44mT:Fe and DpC:Fe complexes. Considering that the studies above demonstrated that both DU145 and HT29 cells respond similarly to NDRG1, experiments with the pharmacological agents only implemented the DU145 cell-type (Figure 9E). DU145 cells were incubated for 24 h/37°C with control medium, DFO (250 μM), Dp44mT (5 μM), DpC (5 μM), DFO:Fe (1:1; 250 μM), Dp44mT:Fe (2:1; 5 μM), DpC:Fe (2:1; 5 μM), FeCl_3_ (250 μM), or Dp2mT (5 μM). These incubation conditions with DFO, Dp44mT and DpC have been previously demonstrated to efficiently induce NDRG1 expression [56, 60, 62]. Furthermore, we have shown that Dp44mT uptake by cells saturates at 5–10 μM [63]. At this concentration, the level of agent is pharmacologically relevant in humans, as the structurally-related thiosemicarbazone, Triapine, has been observed in the serum at similar levels [64, 65]. The much higher concentration of DFO used, relative to Dp44mT and DpC, was due to the fact that this former agent does not readily permeate cell membranes, and thus, far greater concentrations are required to bind cellular iron [29, 57, 60, 66].

Assessing the effect of the agents on NDRG1 expression, DFO, Dp44mT and DpC markedly and significantly (*p* < 0.001) increased NDRG1 expression relative to the control in DU145 cells (Figure 9E). In contrast, the controls which cannot bind cellular iron, namely Dp2mT or the DFO:Fe complex, had no significant (*p* > 0.05) effect on NDRG1 expression. Incubation of DU145 cells with the Dp44mT:Fe or DpC:Fe complexes also resulted in significant (*p* < 0.001–0.01) increases in NDRG1 expression relative to control cells, suggesting that ROS generation by these compounds may also play a role in regulation of NDRG1 expression.

Incubation of cells with DFO, DFO:Fe, FeCl_3_, or Dp2mT did not significantly (*p* > 0.05) alter the phosphorylation of c-Src at Tyr416 or total c-Src compared to the untreated control (Figure 9E). These findings suggest that since DFO could increase NDRG1 expression, chelation of iron alone and up-regulation of NDRG1 is not sufficient to decrease c-Src phosphorylation at Tyr416. In contrast, incubation with Dp44mT, DpC, Dp44mT:Fe, or DpC:Fe, significantly (*p* < 0.001–0.01) decreased the phosphorylation of c-Src at Tyr416 relative to the control, with no significant (*p* > 0.05) alteration being observed with total c-Src levels in DU145 cells (Figure 9E). These studies indicate that novel thiosemicarbazones with potent redox and anti-tumor activity (*i.e*., Dp44mT and DpC) can inhibit c-Src through decreased phosphorylation at Tyr416.

## DISCUSSION

**Figure 10:**
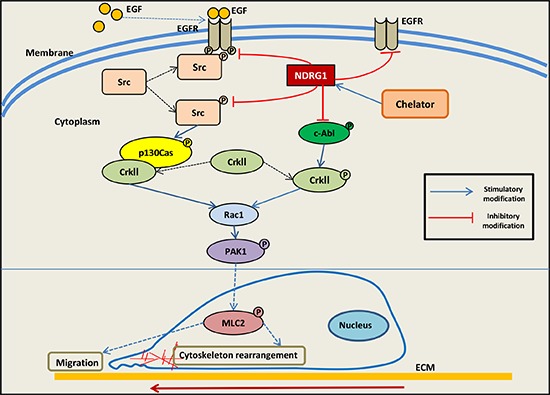
Schematic illustration summarizing the EGFR-c-Src-Rac1 pathway and the inhibitory effect of NDRG1 on cell migration as demonstrated in this investigation NDRG1 expression inhibits c-Src phosphorylation at its activating site (Tyr416). This occurs through NDRG1-induced reduction in EGFR expression, abrogation of EGF-mediated EGFR activation, and thus preventing the EGFR-c-Src interaction. Moreover, NDRG1 is shown to suppress Rac1 activity by modulating the phosphorylation of a c-Src downstream effector, namely p130Cas and its association to CrkII, which acts as a molecular switch to activate Rac1. Additionally, NDRG1 also affected another signaling molecule involved in modulating Rac1 signaling, namely c-Abl activation, which inhibited the phosphorylation of CrkII which is required for activation of Rac1 signaling.

Recent studies have demonstrated that NDRG1 acts as a metastasis suppressor with its expression correlating with the inhibition of cancer progression *in vivo* and cancer cell migration and invasion *in vitro* [21, 28–31, 56, 67–69]. However, the precise molecular mechanisms underlying these anti-metastatic effects of NDRG1 are not fully understood. Herein, we deciphered a novel mechanism by which NDRG1 mediates its inhibitory functions on cancer cell migration in prostate and colorectal cancer cells. In the current investigation, we demonstrate that NDRG1 expression has a unique role in decreasing c-Src activation by reducing both EGFR expression and activation, as well as its binding to c-Src. Moreover, NDRG1 inhibited Rac1 activation downstream of c-Src through down-regulating p130Cas phosphorylation, which prevents p130Cas-CrkII complex formation, and thus, Rac1 activation (Figure 10). Additionally, NDRG1 also decreased the phosphorylation and activation of c-Abl, resulting in the suppression of CrkII phosphorylation which occurred through a mechanism independent of the inhibition of c-Src (Figure 10). Hence, significantly, this is the first report demonstrating that NDRG1 compromises c-Src activation by modulating EGFR and its interaction with c-Src. Subsequently, this effect attenuates downstream signaling of c-Src, namely by inhibiting the p130Cas-CrkII-Rac1 pathway, leading to inhibition of cancer cell migration.

c-Src is a non-receptor tyrosine kinase that is de-regulated in multiple cancers, and aberrant c-Src signaling contributes to diverse aspects of tumor development, including proliferation, survival, adhesion, migration, invasion and metastasis [3, 4]. In fact, in addition to the effects of NDRG1 on inhibiting oncogenic c-Src signaling through EGFR and Rac1 reported herein, it was recently reported that the metastasis suppressor, KAI1/CD82, exerts its anti-oncogenic function in prostate cancer cells by inhibiting CDCP1-mediated enhancement of c-Src activity, leading to reduced HIF-1α and VEGF expression [70]. Moreover, KAI1/CD82 was demonstrated to decrease the activity of c-Src and its substrate p130Cas, as well as receptor tyrosine kinase c-Met activity [71]. However, the inhibition of c-Src activity by KAI1/CD82 was independent of c-Met [71]. In contrast, the present investigation revealed that NDRG1 expression decreased the level of EGFR and abrogated EGF-induced EGFR activation, leading to decreased c-Src activation regardless of EGF treatment (Figure 2A, 2B; Supplementary Figure 1B, 1C). Furthermore, immunoprecipitation studies showed that NDRG1 prevented the coupling of EGFR to c-Src (Figure 2C). Hence, these novel findings revealed that NDRG1 affects the interaction of EGFR and c-Src, which is recognized as a critical mechanism involved in up-regulating c-Src activity [42]. Collectively, it can be concluded that the modulation of the oncogenic activity of c-Src by metastasis suppressors is the result of multiple mechanisms. Herein, for the first time, we demonstrate that NDRG1 plays an important role in suppressing c-Src activation through a unique EGFR-associated process (Figure 10).

As a major target of c-Src signaling, p130Cas has been demonstrated to participate in signaling events that control cell migration by regulating actin rearrangement, cell adhesion and membrane ruffling [11]. Recent evidence suggests that the coupling of p130Cas and unphosphorylated CrkII induces DOCK180 recruitment to the complex, and consequently, this activates Rac1 resulting in signaling events leading to actin re-organization [53]. Therefore, p130Cas and CrkII coupling provides a molecular switch that modulates cell migration [45]. Importantly, the formation of the p130 Cas-CrkII complex is initiated by p130Cas phosphorylation induced by c-Src and/or other protein tyrosine kinases [11]. As discussed earlier, c-Src activation was markedly attenuated by NDRG1 expression (Figure 1B, C and Supplementary Figure 1A), and resulted in the following cascade of events: (1) decreased phosphorylation and activation of p130Cas; (2) decreased association of p130Cas with CrkII; and (3) a decrease of activated Rac1. Moreover, silencing c-Src expression or inhibiting c-Src activation, reversed the elevation of p130Cas phosphorylation and Rac1 activation upon NDRG1 silencing. This result further confirmed the role of NDRG1 in suppressing c-Src activation as well as its downstream signaling cascades. Of note, the metastasis suppressor, KAI1/CD82, has also been shown to down-regulate the coupling of p130Cas and CrkII by decreasing total p130Cas levels, but not its phosphorylation [72], which differs from the mechanism implemented by NDRG1 reported herein.

The small GTPase Rho family member, Rac1, behaves as an important regulator of multiple facets of cell motility, ranging from lamellipodia formation, assembly of focal adhesions and membrane protrusions to the generation of stress fibers, by either directly acting on the cytoskeleton, or by interacting with the above signaling molecules [73]. In fact, Rac1 plays a critical role in terms of bridging signaling from the c-Src-p130Cas-CrkII pathway to the cytoskeleton [45]. We demonstrate that under the influence of NDRG1, Rac1 activation is markedly reduced, and this concurs with the fact that: (1) c-Src activity is tightly related to Rac1 activation [18], and (2) that the p130Cas-CrkII association is the molecular switch that stimulates Rac1 activity [13].

Being a direct effector of Rac1, activated PAK1 is involved in the formation of lamellipodia, filopodia and stress fibers, which are necessary for cell migration and invasion [48]. Several signaling molecules are implicated in PAK1-induced cytoskeletal structure reorganization, including MLC2 [74]. It has been shown that PAK1 phosphorylates and activates MLC2, leading to cell motility [75]. Interestingly, our previous studies demonstrated that NDRG1 substantially inhibited tumor cell migration by reducing ROCK/pMLC2 pathway activation [31], which concurs with the findings from the current investigation. Moreover, we demonstrated in this study, that the inhibition of cell migration upon NDRG1 expression occurs through a mechanism involving suppression of Rac1 and PAK1 activation. Hence, the molecular mechanisms underlying the effect of NDRG1 involves inhibition of PAK1 through the c-Src pathway, leading to decreased pMLC2 that reduces cell motility (Figure 10).

CrkII is a crucial element of the p130Cas-CrkII complex, with phosphorylation of CrkII at Tyr221 forming an intra-molecular interaction *via* its SH2 domain that prevents its interaction with activated p130Cas and DOCK180 [51]. Herein, immunoblot analysis revealed that while NDRG1 expression decreased CrkII phosphorylation (Figure 6), it also reduced p130Cas phosphorylation (Figures 3, 7, 8), which led to decreased levels of the CrkII-p130Cas complex (Figure 3C), with the latter being the likely mechanism that led to the NDRG1-dependent decrease in Rac1 activation (Figure 4).

Interestingly, there is evidence that phosphorylation of CrkII at Tyr221 does not lead to simple “on-and-off” signaling, but is rather a context-dependent and dynamic process that regulates Rac1 activation and membrane relocation by a non-canonical mechanism [15, 53, 76]. Indeed, the Tyr221 phosphorylation of CrkII may be involved in redirecting signaling from p130Cas-CrkII complex to an alternative signaling pathway [77]. Considering the possible regulation of these effects by NDRG1, the observed inhibition of CrkII Tyr221 phosphorylation by NDRG1 in this study would additionally be expected to prevent activation of Rac1 by this non-canonical signaling pathway.

Currently, NDRG1 is being considered as an important oncogenic target of a new group of potent anti-tumor chemotherapeutics belonging to the DpT class, which include Dp44mT and DpC [55–58]. These agents markedly up-regulate NDRG1 through HIF-1α-dependent and -independent mechanisms [55, 78] and have been demonstrated to block the EMT and cell migration *in vitro* [29, 31], and inhibit the growth of a variety of belligerent solid tumors by both the oral and intravenous routes [33, 57–59]. Moreover, Dp44mT has been shown to markedly suppress tumor metastasis *in vivo* [28]. The dissection of the activity of these agents requires a thorough analysis of the molecular effector mechanisms of NDRG1 and the present study has clearly demonstrated its effect on a major proto-oncogene, namely c-Src. We have demonstrated that both Dp44mT and DpC act to markedly increase NDRG1 expression, but also inhibit the activation of c-Src, suggesting the therapeutic efficacy of these agents involves, at least in part, the suppression of this proto-oncogene. At present, DpC is under active preclinical development and clinical trials are planned for 2015. Hence, the addition of these agents to the current chemotherapeutic armamentarium will be important for combating metastasis, which is a major cause of cancer mortality.

In summary, this investigation highlights a novel mechanism mediated by NDRG1 in inhibiting cancer cell migration. These studies demonstrate that NDRG1 modulates c-Src activation which is achieved by down-regulation of both the expression and EGF-induced activation of EGFR, preventing its interaction with c-Src. Moreover, NDRG1 attenuates the downstream signaling of c-Src which involves the p130Cas-CrkII-Rac1 pathway, and this leads to suppression of cellular migration. Therefore, NDRG1 inhibits c-Src oncogenic activation as well as its downstream signaling to exert its anti-metastatic activity.

## MATERIALS AND METHODS

### Cell culture/treatments

Human prostate and colon cancer cell lines, DU145 and HT29 (American Type Culture Collection; Manassas, VA), were grown under established conditions [29]. NDRG1 over-expressing and silenced clones of the DU145 and HT29 cells were generated as described previously [29, 79]. Human prostate cancer cells, PC3MM, were stably transfected with tetracycline (TET)-inducible (TET-ON) human NDRG1 (pcDNA5/TO/Flag-Drg-1) and kindly provided by Dr K. Watabe (Southern Illinois University School of Medicine, USA) [79]. These cells were grown in RPMI-1640 medium (Life Technologies) supplemented with 10% (v/v) fetal bovine serum (FBS; Sigma-Aldrich), penicillin (100 IU/mL), streptomycin (100 μg/mL), glutamine (2 mM), non-essential amino acids (100 mM) and sodium pyruvate (100 mM; all supplements from Life Technologies). All cells were grown at 37°C in 5% CO_2_ in a humidified incubator.

Human recombinant EGF was obtained from Cell Signaling Technology (Cat.#:8916, Boston, MA) and used at a final concentration of 10 ng/mL or 50 ng/mL for HT29 and DU145 cells, respectively. For EGF treatment, the cells were incubated in serum-free medium overnight, and then incubated with EGF for 5 min for HT29 cells or 10 min for DU145 cells, respectively. These incubation conditions were chosen based upon optimization experiments.

The thiosemicarbazones, Dp44mT, DpC, and Dp2mT, were synthesized and characterized using standard methods [80, 81], while DFO was purchased from Novartis (Basel, Switzerland). Dp44mT, DpC and Dp2mT were dissolved in DMSO at 10 mM and then diluted in media containing 10% (v/v) FBS so that the final [DMSO] was ≤ 0.1% (v/v). The iron complexes of Dp44mT, DpC and DFO were prepared by the addition of FeCl_3_. Since both Dp44mT and DpC are tridentate, the ligand: iron ratio implemented was 2:1, while as DFO is hexadentate, a ligand: metal molar ratio of 1:1 was utilized. Cells were incubated with these agents for 24 h at 37°C.

### Protein extraction/immunoblots

Preparation of cell lysates and immunoblot analysis was performed *via* established protocols [82]. Anti-human primary antibodies were implemented at a 1:1, 000–2, 000 dilution and include: anti-NDRG1 (Cat#:ab37897) from Abcam (Cambridge); anti-Src (Cat#:2123), anti-phospho-Src Family (Tyr416; Cat.#:6943), anti-phospho-Src(Tyr527; Cat.#:2105), anti-p130Cas (Cat.#:13846), anti-phospho-p130Cas (Tyr249; Cat.#:4014), anti-phospho-p130Cas (Tyr410; Cat.#:4011), anti-EGF Receptor (Cat.#:2926), anti-phospho-EGF Receptor (Tyr1148; Cat.#:4404), anti-c-Abl (Cat.#:2862), anti-phospho-c-Abl (Tyr245; Cat.#:2861), anti-PAK1 (Cat.#:2602), anti-phospho-PAK1 (Thr423; Cat.#:2606), anti-PTP-PEST (Cat.#:4864), anti-PTP1B (Cat.#:5311), anti-CrkII (Cat.#:3492) and anti-phospho-CrkII (Tyr221; Cat.#:3491) were from Cell Signaling Technology (Boston, MA); and anti-Rac1 (Cat.#:05–389) was from Millipore (Darmstadt). The secondary antibodies (1:10, 000 dilution) included: anti-goat (Cat.#:A5420), anti-rabbit (Cat.#:A6154) and anti-mouse (Cat.#:A4416) antibodies from Sigma-Aldrich. β-actin (1:10, 000; Cat.#:A1978, Sigma-Aldrich) was used as a loading control.

### Gene silencing by siRNA

Knockdown of c-Src expression using c-Src siRNA was performed following the manufacturer's instructions. Briefly, at ~60–70% confluence, NDRG1-silenced and sh-control cells were transfected with c-Src Select Silencer^®^ siRNA duplexes (Ambion), or the negative control siRNA at 10 nM for 72 h/37°C, using Lipofectamine RNAi MAX^®^ (Invitrogen).

### The c-Src pharmacological inhibitor

The c-Src specific pharmacological inhibitor, (3Z)-N, N-dimethyl-2-oxo-3-(4, 5, 6, 7-tetrahydro-1H-indol-2-ylmethylidene)-2, 3-dihydro-1H-indole-5-sulfonamide (SU6656), was purchased from Millipore (Darmstadt), dissolved in dimethyl sulfoxide (DMSO) and used at 10 μM in culture media (final [DMSO]: ≤ 0.1%(v/v)). This concentration was implemented based on preliminary studies examining the efficacy of the agent *in vitro*.

### Immunoprecipitation

Immunoprecipitation was performed using Dynabeads^®^ protein A/G (Thermofisher) by an established method [30], and appropriate proteins were detected by immunoblot analysis as indicated in figures.

### Rac1 activation assay

Rac1 activity was measured using a specific pull-down assay (Millipore, Cat.#:17–10393) by which the GTP-bound Rac1 was affinity precipitated with the p21-binding domain (PBD) of PAK1. This protein was provided as a fusion to GST conjugated to glutathione-magnetic beads. Briefly, cells were lysed in Mg^2+^ lysis/wash buffer and centrifuged for 5 min at 14, 000 g/4°C. The supernatant was incubated at 4°C with recombinant PBD for 45 min. Samples were washed three times with ice-cold PBS and bound proteins were analyzed by western blot.

### xCELLigence real-time cell migration assay

Cell migration assay were performed by using modified 16-well plates and xCELLigence^®^ DP system (CIM-16, Roche Diagnostics GmbH, Mannheim), which is an electrical impedance-based system that allows for real-time measurement of cell migration [54]. Briefly, after serum starvation for 24 h, 2 × 10^4^ cells were seeded into each upper chamber in serum free medium, while the lower chamber contained 10% FBS medium. Then, for the migration assay, the CIM-16 plate was prepared and locked into the real-time cellular analysis device at 37°C in a 5% CO_2_/humidified incubator. Each condition was performed using a programmed signal detection schedule that was recorded every 15 min for 24 h.

### Statistical analysis

Data are expressed as mean ± SD of at least 3 independent experiments. Student's *t*-test and ANOVA (Graphpad Prism 5.0; GraphPad Software, San Diego, CA) were used with *p* < 0.05 being considered significant.

## SUPPLEMENTAry FIGURES


