# Supplementary material for: The proto-oncogene c-Src and its downstream signaling pathways are inhibited by the metastasis suppressor, NDRG1
Source: Oncotarget. 2015 Apr 10;6(11):8851–74. doi: 10.18632/oncotarget.3316 (PMC4496188; doi:10.18632/oncotarget.3316)
Supplement: Supplementary file 1 [file oncotarget-06-8851-s001.pdf]

SUPPLEMENTARY FIGURES

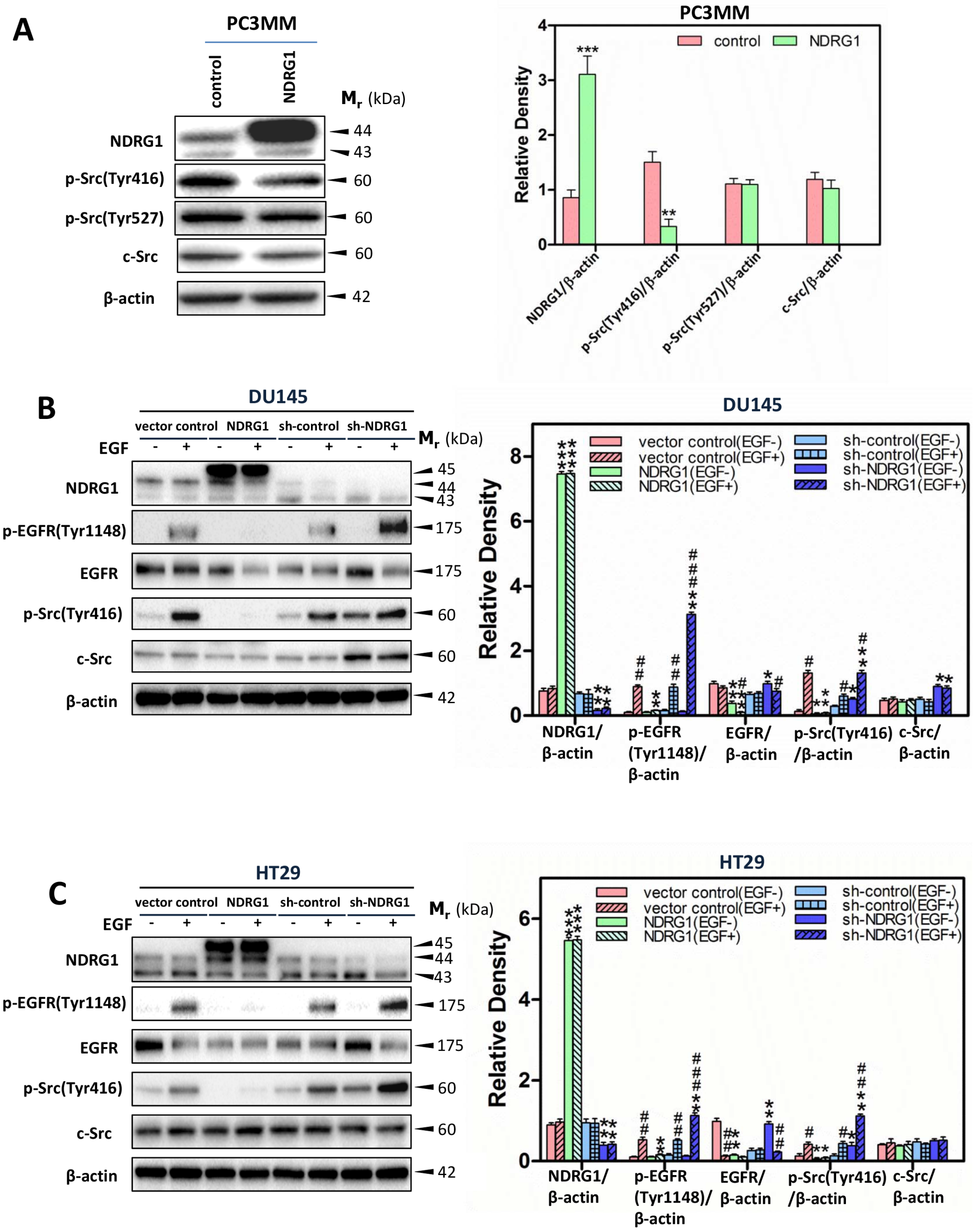

Supplementary Figure 1: NDRG1 inhibited the activation of c-Src in the prostate cancer cell line PC3MM (A) and abrogated EGF-induced EGFR activation and EGFR-mediated c-Src activation (B) DU145 and (C) HT29 cells. (A) Immunoblotting was performed as described in the legend for Figure 1 in PC3MM cells, to examine c-Src phosphorylation (Tyr416 and Tyr527) and total c-Src; (B, C) DU145 and HT29 cells were incubated in the presence or absence of EGF 50 ng/mL/10 min and 10 ng/ml/5 min, respectively. Immunoblotting demonstrating that in (B) DU145 cells and (C) HT29 cells, NDRG1 expression abrogated EGF-induced EGFR phosphorylation (Tyr1148) and c-Src activation (Tyr416). Immunoblotting results are representative of 3-5 experiments. Densitometric data are mean ± S.D. (3–5 experiments); *$p < 0.05$; **$p < 0.01$; ***$p < 0.001$, relative to control cells; $^{\#}p < 0.05$; $^{\#\#}p < 0.01$; $^{\#\#\#}p < 0.001$, relative to vector control or sh-control cells without EGF treatment, as appropriate.

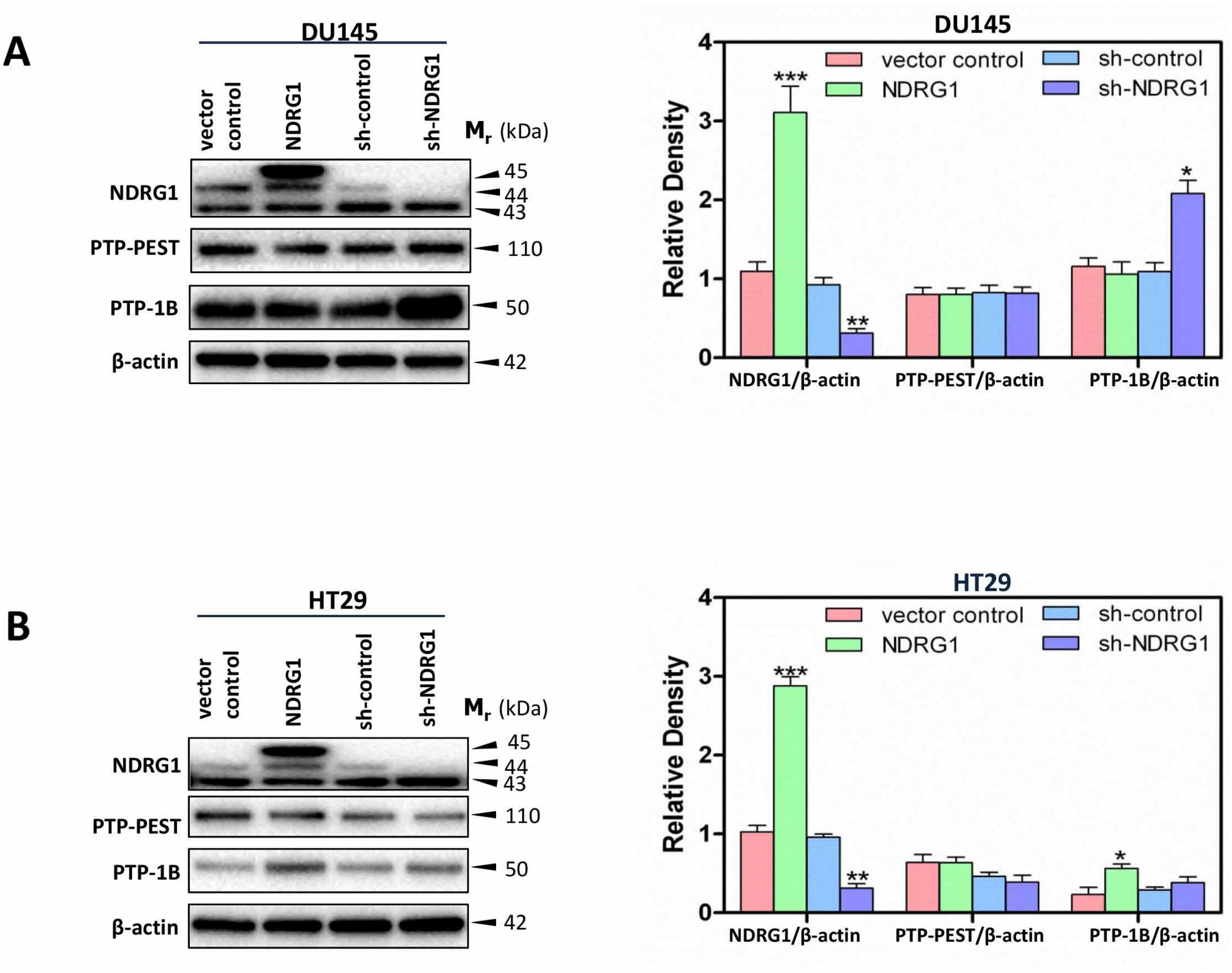

Supplementary Figure 2: NDRG1 expression had no consistent effect on PTP-PEST and PTP-1B expression in (A) DU145 and (B) HT29 cells. Immunoblotting results are representative of 3-5 experiments. Densitometry data are mean ± S.D. (3–5 experiments); $*p < 0.05$; $**p < 0.01$; $***p < 0.001$, relative to vector control or sh-control cells, as appropriate.

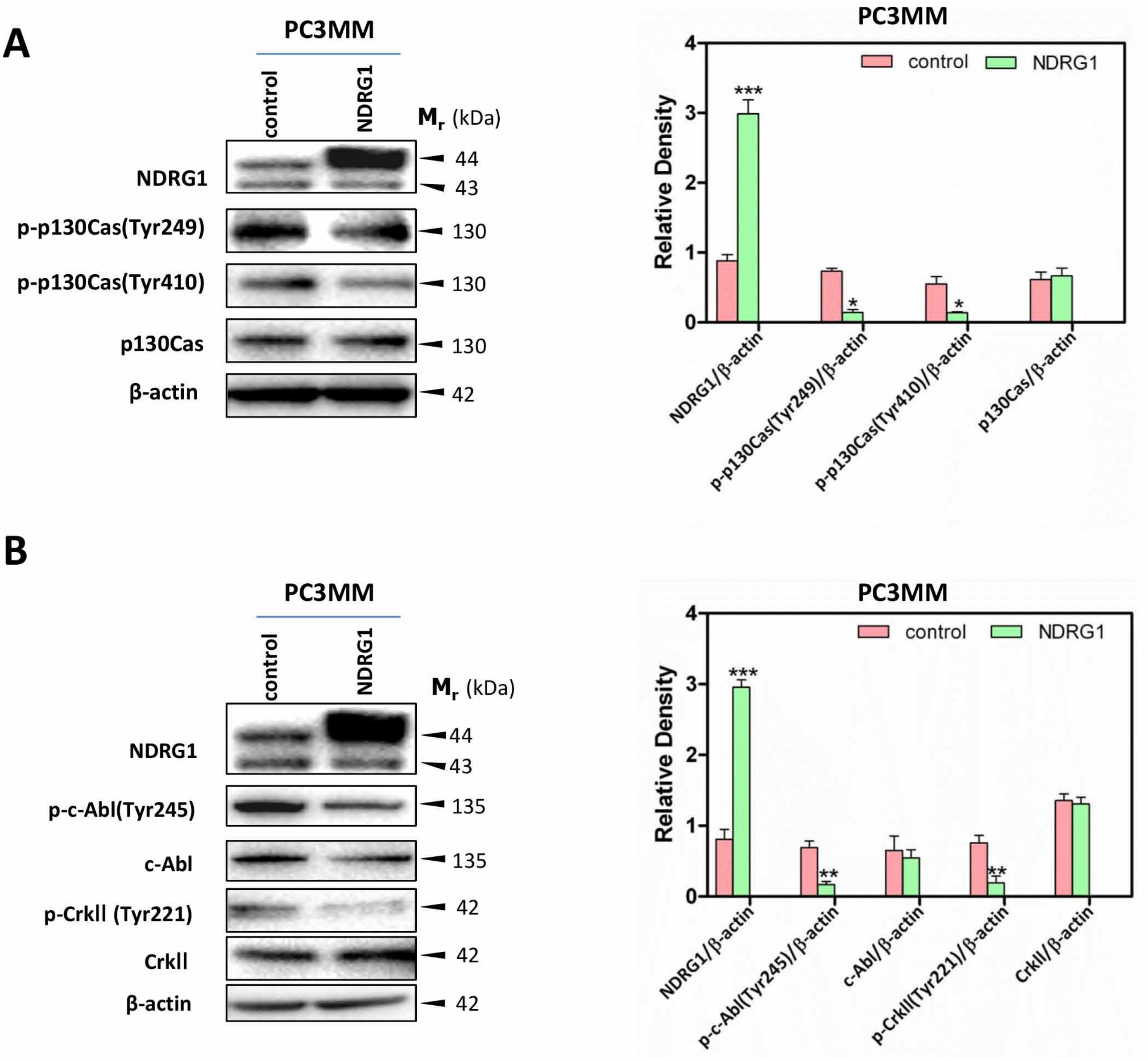

Supplementary Figure 3: NDRG1 inhibited the activation of p130Cas and c-Abl in the prostate cancer cell line PC3MM (A, B) (A) Immunoblotting was performed in PC3MM cells to examine p130Cas phosphorylation (Tyr249 and Tyr410) and total p130Cas; and (B) c-Abl phosphorylation (Tyr245), total c-Abl, p-CrkII (Tyr221) and total CrkII. Immunoblotting results are representative of 3-5 experiments. Densitometry data are mean ± S.D. (3–5 experiments); *$p < 0.05$; **$p < 0.01$; ***$p < 0.001$, relative to vector control or sh-control cells.
